# Progress in Medicine

**Published:** 1898-08-27

**Authors:** 


### Progress in Medicine.

### FEVERS.

(*Continued from page* 354.)

**Small-pox.**—The *Lancet*[17] has insisted that, if vaccination is a matter of State concern, there is no reason for dealing with vaccination and omitting to deal with re-vaccination. It argues that the recognition of this point in the Bill is of the highest importance, whether the operation be made compulsory on parents or not. The administration of the law by the sanitary authorities and not by boards of guardians, and some mode of getting more efficient vaccination than the one vesicle system, are among the needs of the case. The *British Medical Journal* and the Epidemiological Society endorse this view, and the council of the Association has issued an elaborate tract and a brief leaflet setting forth the facts about vaccination, which cannot be too widely read. They are printed at length in the journal.[18] The work of isolating and cultivating the virus of vaccinia has steadily advanced. It will be recollected that Copeman succeeded in growing it in a pure form by inoculating hen's eggs, and that these cultures when passed through calves produced normal vesicles in children. Stanley Kent,[19] who showed specimens of Klein's diplo-bacilli in 1894, has been able to demonstrate them in glycerinated lymph after keeping, and further, to grow them in a mixture of egg albumen and glycerine in a pure state, and to make sub-cultures of them from tube to tube. These organisms are found then in the tissues about the vesicles, and in the lymph, can be grown outside the body, and reproduce the vesicles when inoculated into calves.

Copeman,[20] in his Milroy lectures, puts forward the view that variola and vaccinia have both arisen from a common ancestor, but that the germ of small-pox is a variety which has been rendered more virulent by passing through many generations of human beings. When inoculated into calves, as Copeman and others have done successfully, it tends to revert to an ancestral condition, and becomes vaccinia. This type is a more permanent one, and shows little tendency to "sport" or to produce a generalised eruption, though so nearly allied to the specialised form called variola that it protects against it. The writer's cultivations of the germ in eggs, though completely successful at first, are difficult to repeat, and he has hitherto failed in repeating Freyer's inoculation of the gland serum from previously vaccinated animals. He acknowledges the presence of the protozoa described by Guarnieri and others during the invasion of these diseases, but holds that they have no pathological importance, while the bacilli which he himself obtained could be transferred from calf to calf, and produced normal vesicles, besides protecting the animals from subsequent vaccination. Indeed, after several transmissions they were successfully used for human vaccination. His paper concludes with a full account of the mode of preparing glycerinated lymph, and its great advantages. A remarkable contribution to the vaccination controversy is given in the statement issued by the Health Office, Berlin,[21] showing the complete success of vaccination in Germany. Only one death has taken place in the army in 24 years, and the average rate in the whole population is now only 2·5 per million, in spite of frequent infection over the frontiers. The cause that led to the severe vaccination laws was the great outbreak which occurred during the French war of 1870. France lost 23,000 soldiers by small-pox, or altogether 90,000 persons, and the mortality in Germany was also considerable, though in the comparatively well vaccinated German army only 278 soldiers perished. In Russia so late as 1891-3 288,000 persons died of small-pox. The anti-vaccinators seek to deny the value of even the exact German army statistics and the increased stringency of the German law of 1874, in spite of the recorded facts, but the results of the German law are unassailable. The cause of the cessation of the Gloucester epidemic was by the anti-vaccinators attributed to rain and other sanitary agents, but the Lancet[22] remarks that it could not be due to flushing the drains by the August rains, for the epidemic had already come to an end, nor to the new water supply, for that, too, was not introduced till later, nor to new drainage, for there was none, nor to the removal of insanitary jerry-built houses, for these remained. Yet 35,000 people were left unattacked, and it is remarkable that this was the number within a few hundreds of the persons who were vaccinated or revaccinated at the time of the epidemic. This then was the barrier which saved a very great proportion of the 35,000 from taking small-pox. No less than 26,000 were revaccinated in the seven months.

In reference to the theory that isolation hospitals may profitably be substituted for vaccination, it is pointed out that 750,000 beds would be needed for the United Kingdom,[23] and the cost of providing them would amount to no less than 150 millions of money besides the cost of maintenance, repair, and the wages of the quarantined members of families where cases of small-pox had occurred.

The late Middlesbrough epidemic[24] produced 1,200 cases, with a mortality of 153, or only 12·7 per cent., while at Gloucester the deaths were 24·9 per cent. The mortality of the unvaccinated in the two towns was respectively 46·6 and 40·8, and that of the vaccinated 8·2 and 9·5. The majority of the cases in Middlesbrough were in adults or adolescents who had been vaccinated only during infancy, while at Gloucester the numbers were swelled by the vast number of unvaccinated children who were attacked. The infant population of Middlesbrough was protected, and escaped, while the incidence on adults shows the danger of neglecting revaccination. Each epidemic thus leaves a useful lesson behind it.

**Scarlatina.**—M. P. Myers[25] notices a hitherto unobserved symptom in scarlatina. This consists in a numbness or tingling sensation in the hands and feet, especially in the palms and tips of the fingers. Occasionally it amounts to a slight paralysis of the extremities. It appears with the eruption and sometimes before it. The duration varies from a few minutes to several days. It is to be distinguished from the itching of the eruption, from the tumefaction produced by the same cause, and from the effects of articular rheumatism. The author has not observed this symptom in other eruptions. The mode of entrance of the poison of this disease is no doubt usually by the fauces, but Washbourn[26] points out that cases of a mild type are often seen where the fauces are normal, but where a sore or surgical wound is present, and that patients suffering from extensive burns are specially liable to attacks. Though the evidence is not conclusive, there is much reason to think that the poison in these cases has entered through the wound. Whether the germs after entering the body remain localised or spread through the tissues is unknown, but there does not appear to be any evidence of their diffusion, not even of their presence in the skin. Indeed, the end of desquamation is not an absolutely reliable proof that infectiousness has ceased. Two types of fatal cases are to be distinguished—S. maligna is due to a profound intoxication, and no bacteria are found in the blood or organs. On the other hand, in S. anginosa the fauces are swollen and covered with exudation. There is extensive ulceration and infiltration of the surrounding tissues. In this class of cases vast quantities of streptococci are present, but the author has had no success with the serum prepared for them. As to other mixed infections, it is noticeable that patients are very liable to infection with diphtheria, which spreads rapidly in a fever ward.

Seitz[27] remarks on the great variations of the mortality of the disease. In different years he has seen the death-rate range from 2·6 to 15 per cent. Children in their first year, though not so often attacked as their elders, showed 40·6 per cent. of deaths. He only found two cases of true second attacks in 833 persons, while a return of the rash after a week or so was not infrequent. Nephritis depended rather on the type of the epidemic than on the weather or the diet, and the worst forms were seen even in children in bed on a milk diet. He does not, however, omit such precautions on account of their occasional failure. The false membranes in the throat were not usually due to Loeffler's bacillus, which he found only seven times in 98 cases examined. Harold Lowe[28] reports a very severe case of scarlatina, with hæmorrhagic septicæmia treated by the streptococcal serum, in which recovery followed. Suppuration of the middle ear was also present, and the mastoid antrum was opened surgically. The whole body was at one time covered with purpuric spots, and the condition was extremely grave, not only from the original disease, but also from epistaxis. Cultivations showed the presence of streptococci in the blood, and 263 c.c. of the serum were used. Mr. Ballance holds that it certainly saved the patient's life, and remarks on the value of the continued injections, even when the patient was almost moribund.

[17] Lancet, Mar. 26. [18] Brit. Med. Jour., Mar. 5. [19] Lancet, May 31. [20] Brit. Med. Jour., May 7. [21] Brit. Med. Jour., July 2. [22] Lancet, Mar. 26. [23] Lancet, Feb. 26. [24] Brit. Med. Jour., April 9. [25] New York Med. Jour., Mar. 26. [26] Clin. Jour., June 1. [27] Amer. Jour. Med. Sci., April. [28] Lancet, Mar. 19.

### DISEASES OF THE LUNGS.

**Bronchitis.**—Leech[1] makes some very practical remarks on the treatment of bronchitis by drugs. In acute bronchitis of adults a combination of acetate of ammonia, spirit of nitrous ether, and ipecacuanha or antimony is commonly used, and no better combination can be employed. But an error is usually committed in giving too small a dose of the two first-named drugs. Leech finds that less than two drachms of liquor ammonii acetatis does not act on the skin or give the general relief which often results from a larger dose. He generally begins with three drachms and increases it to six drachms if the skin does not act freely. Again, spiritis ætheris nitrosi, which is often given only in fifteen or twenty mimim doses, should be given in drachm or two drachm doses—smaller doses fail to act as a diaphoretic, It is to be remembered that, mixed with water only, spirit of nitrous ether rapidly decomposes, but this decomposition is rendered much slower by the presence of acetate of ammonia. Antimony in doses of one-twentieth of a grain is of most service where there are abundant small basic moist sounds, whilst ipecacuanha is more useful than antimony when there are dry rhonchi all over the chest with irritable cough.

In young children attacked with acute bronchitis, with a high temperature and a few scattered rhonchi, antipyrin, say five grains in a child five years old, is of more advantage than acetate of ammonia.

In cases of chronic bronchitis, ammonia, senega, squill, and ipecacuanha are the drugs most commonly used, digitalis and strychnine being given when there is evidence of failing or defective cardiac or respiratory organs. Carbonate of ammonia often fails to benefit the patient because it is given in too small a dose; perhaps three or five grains, or ten or twenty minims of aromatic spirit of ammonia, every four hours. Again, the drugs with which ammonia is frequently combined —squill, senega, &c.—are not advantageously given at short intervals. Leech recommends that the carbonate of ammonia should be dissolved in water and the solution given in milk. Five grains may thus be administered every hour or two, according to the nature of the case, in the milk which the patient takes. As a rule the patient does not object to the taste. The senega and squill, to which, if necessary, strychnine may be added, can then be given in larger doses at greater intervals, and they have not then the same tendency to irritate the stomach as when given oftener.

**Pneumonia: Diphtheritic Pneumonia.**—The diphtheritic bacillus does not always remain confined to the diseased mucous membrane, but sometimes invades the blood and internal organs—the lungs, spleen, and lymphatic glands. Of these organs the lungs are the most frequently affected. Thus, in 26 fatal cases, Kanthack and Stephens found the bacillus with ease in the lungs, and in 15 of these broncho-pneumonia was present. As to the relation of the broncho-pneumonia to the bacillus, in almost all cases other organisms, such as streptococci and staphylococci, are found in the lesions; and it has therefore been supposed that the broncho-pneumonia is due to these organisms, and not to the diphtheritic bacilli.

However, Flexner and Anderson[2] have published experiments which show that it may be a true diphtheritic lesion; they injected pure cultures into the tracheas of rabbits, which caused a definite and often widespread pneumonic process. The pneumonia was lobular or pseudo-lobular in character, and for the most part cellular, fibrin playing a relatively small part. In certain cases, however, pneumonia failed to be produced.

**Streptococcic Pneumonia.**—Denny[3] calls attention to a type of pneumonia depending on streptococcic infection. The onset may be like that of ordinary pneumonia, though often it is less sudden. The sputum is more purulent, and less often rusty. The appearance of the patient is characteristic; there is less flushing of the face, and a peculiarly septic look. The temperature is irregular. There is often no crisis, but the temperature comes down by lysis, often after a course of three or four weeks. The physical signs appear late, and the upper lobes are more frequently involved than in the ordinary forms. The local process has a tendency to wander, giving rise to the expression, "Erysipelas of the lungs." Hence the physical signs are of long duration. Thus Wiesmayr reported 39 cases of pneumonia, in 34 of which only pneumococci were found; whilst in five there were streptococci, twice with pneumococci and three times alone. Now, in all the pneumococcic cases, with one exception, resolution took place by the twelfth day, whilst in not a single streptococcic case did this occur before the nineteenth, and in one not before the fortieth day. Pneumococci are very short-lived, and if an active process goes on in the lungs for more than two weeks it is fairly certain that some organism other than the pneumococcus, probably the streptococcus, is present. The diagnosis must be based on finding the streptococcus in the sputum; typhoid fever, influenza, and tuberculosis have to be excluded. The prognosis is better as regards life than ordinary pneumonia, but, as stated, a longer course is to be expected. Treatment by anti-streptococcic serum suggests itself, but no information on this point is available.

**Pneumonia after Injury.**—The most diverse views have been held in reference to the relation of pneumonia to injuries of the chest. Thus Fagge says that true pneumonia is not set up by local injuries to the chest, wounds of the lungs, or foreign bodies in the bronchi, whilst, on the other hand, Osler briefly affirms that pneumonia follows traumatism with great frequency. Harris[4] has recently recorded a case in which there was a pneumonia of the left lower lobe, probably consequent on an injury to the chest, which pneumonia became infected a few weeks before death with tubercle, resulting in a tuberculosis most marked and advanced in the same lobe.

**Treatment of Pneumonia by Salicylic Acid.** — De Becker[5] has treated cases of pneumonia for the last two years by salicylic acid. The guiding symptom should be the expectoration; it should be given until this is free. In the case of children one and a half grains were given every four hours, whilst in adults seven grains was the dose. Cardiac disease and extreme weakness are contra-indications to this treatment.

[1] Leech, Practitioner, May, 1893. [2] Flexner and Anderson, Johns Hopkins Hospital Bulletin. April, 1898. [3] Denny, Boston Med. and Surg. Jour., April 14, 1898. [4] Harris, Lancet, April 16, 1898. [5] De Becker, Ann. et Bull. de la Soc. de Méd. d'Anvers, Feb., 1898.

